# The calcium sensitizer drug MCI-154 binds the structural C-terminal domain of cardiac troponin C

**DOI:** 10.1016/j.bbrep.2018.10.012

**Published:** 2018-11-01

**Authors:** Monica X. Li, Shorena Gelozia, Gaddafi I. Danmaliki, Yurong Wen, Philip B. Liu, M. Joanne Lemieux, Frederick G. West, Brian D. Sykes, Peter M. Hwang

**Affiliations:** a Department of Medicine, University of Alberta, Edmonton, Alberta, Canada T6G 2R3; b Department of Chemistry, University of Alberta, Edmonton, Alberta, Canada T6G 2G2; c Department of Biochemistry, University of Alberta, Edmonton, Alberta, Canada T6G 2H7; d School of Life Science and Technology, Xi’an Jiaotong University, Xi’an, China

**Keywords:** Solution NMR spectroscopy, Calcium sensitizer, Drug binding, Protein-protein interaction

## Abstract

The compound MCI-154 was previously shown to increase the calcium sensitivity of cardiac muscle contraction. Using solution NMR spectroscopy, we demonstrate that MCI-154 interacts with the calcium-sensing subunit of the cardiac troponin complex, cardiac troponin C (cTnC). Surprisingly, however, it binds only to the structural C-terminal domain of cTnC (cCTnC), and not to the regulatory N-terminal domain (cNTnC) that determines the calcium sensitivity of cardiac muscle.

Physiologically, cTnC is always bound to cardiac troponin I (cTnI), so we examined its interaction with MCI-154 in the presence of two soluble constructs, cTnI_1–77_ and cTnI_135–209_, which contain all of the segments of cTnI known to interact with cTnC. Neither the cTnC-cTnI_1–77_ complex nor the cTnC-cTnI_135–209_ complex binds to MCI-154. Since residues 39–60 of cTnI are known to bind tightly to the cCTnC domain to form a structured core that is invariant throughout the cardiac cycle, we conclude that MCI-154 does not bind to cTnC when it is part of the intact cardiac troponin complex. Thus, MCI-154 likely exerts its calcium sensitizing effect by interacting with a target other than cardiac troponin.

## Highlights

• MCI-154 is a small molecule calcium sensitizer in cardiac muscle.
• The N-domain of cardiac troponin C controls calcium sensitivity in cardiac muscle.
• MCI-154 binds weakly to the promiscuous C-terminal domain of troponin C.
• Cardiac troponin C does not bind MCI-154 in the presence of troponin I.
• MCI-154 does not exert its calcium sensitizing effect directly through troponin C.

## 1 Introduction

In heart failure, cardiac output is insufficient to satisfy the metabolic needs of the body. In acute decompensated heart failure, cardiac output becomes so compromised that, unless something is done imminently, clinical deterioration and death will ensue. Positive inotropes such as dobutamine and milrinone can increase cardiac output, but these are also associated with systemic hypotension, arrhythmias, and increased mortality [1].

Dobutamine and milrinone activate β_1_-adrenergic signaling pathways via direct binding to G-protein-coupled receptors in the case of dobutamine or dual phosphodiesterase-3 and − 4 inhibition by milrinone [2], [3]. This leads to increased cyclic AMP concentrations and activation of protein kinase A, which phosphorylates many different targets within the cardiac myocyte including L-type calcium channels and phospholamban, which regulates sarcoplasmic reticulum Ca^2+^-ATPase (SERCA) [4]. Increased calcium fluxes enhance cardiac output, but at the expense of increased myocardial oxygen consumption and increased risk of arrhythmias. An alternative approach is to use “calcium sensitizers”, drugs that impact muscle function not by altering calcium fluxes, but by enhancing the contractile response to calcium. The best known cardiac calcium sensitizer is levosimendan, which has undergone many trials for the treatment of acute decompensated heart failure [5].

Many calcium sensitizers are purported to act on the cardiac troponin complex (cTn), the key calcium dependent switch that turns muscle contraction on and off with every heartbeat. It consists of three protein subunits: C, I, and T. The calcium dependence of the complex derives from troponin C (cTnC), which has two calcium-binding EF-hand domains. The N-terminal regulatory domain (cNTnC) determines the contractile state of the heart. Its calcium affinity is tuned to calcium fluctuations in the cardiac myocyte, so that it becomes significantly occupied only during systole, when the cytoplasmic free calcium concentration rises to about 1 μM (for a review, see [6]). Calcium binding stabilizes the open conformation of cNTnC, allowing it to bind the switch region of cardiac troponin I (cTnI_147–163_) [7]. This removes adjacent inhibitory cTnI segments from actin, favoring activation of actin-myosin ATPase and cardiac muscle contraction.

**Fig. 1:**
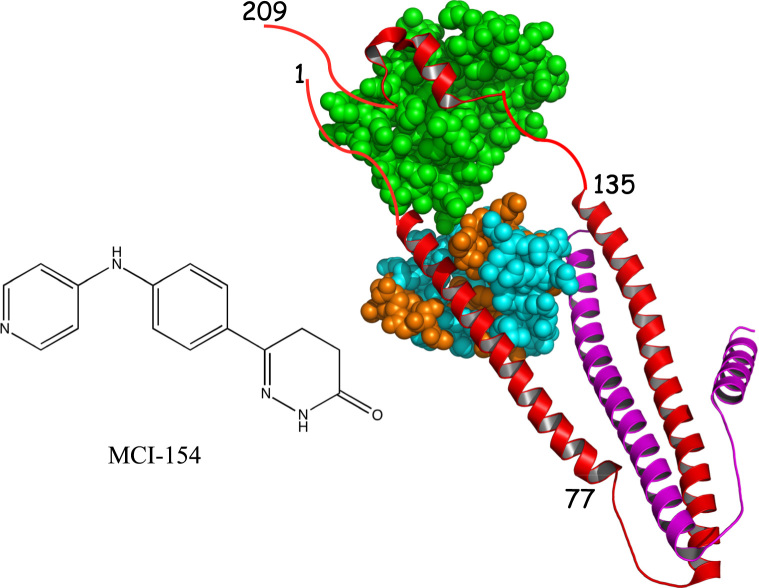
Chemical structure of MCI-154; and structural frame of the cardiac troponin complex (pdb 4Y99) (cTnC in spheres, cTnI in red, and cTnT in magenta). The MCI-154 binding site is identified by coloring in orange the residues that show the greatest chemical shift perturbations upon titration of cTnC•3Ca^2+^ with MCI-154 (see Fig. 2). The figure was created by PyMOL.

In theory, calcium sensitizers could act by stabilizing the calcium-bound activated form of cNTnC. This has been convincingly demonstrated for bepridil, a drug originally designed to treat angina by blocking calcium channels, but serendipitously shown to also act as a calcium sensitizer [8]. It was later demonstrated that bepridil binds cNTnC to stabilize its calcium-bound open conformation [9]. Bepridil, however, is not an effective troponin activator because it displaces cTnI switch peptide from cNTnC [10]. We thus searched for compounds that bind to cNTnC without displacing cTnI switch peptide and discovered that derivatives of diphenylamine are effective for this purpose [11]. A structurally similar compound is MCI-154 (Fig. 1), which was previously developed by Mitsubishi Chemical Corporation as a calcium sensitizing agent. It was shown to increase the calcium sensitivity of cardiac myofibrils as well as isolated cardiac troponin C [12], [13]. Thus, out of all the calcium sensitizers developed to date, MCI-154 had some of the strongest evidence for a mechanism of calcium sensitization through direct binding to cardiac troponin C [14]. We thus set out to more thoroughly investigate the interaction of MCI-154 with cTnC.

## 2 Materials and methods

### 2.1 Synthesis of MCI-154 (Scheme 1)

**Scheme 1:**
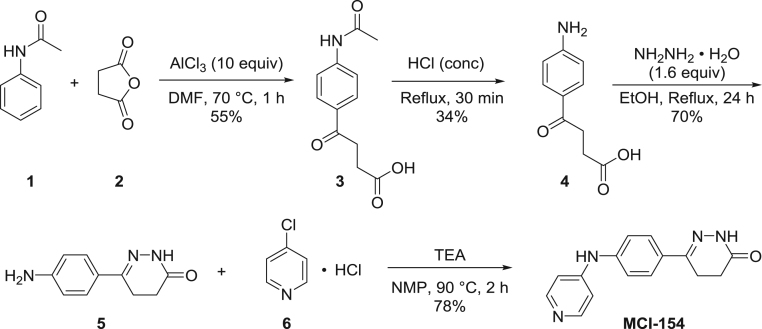
Synthesis of MCI-154.

Synthesis of 6-(4-(pyridin-4-ylamino)phenyl)-4,5-dihydropyridazin-3(2H)-one (MCI-154) was performed by following the synthetic route outlined on Scheme 1. Intermediate 4-(4-acetamidophenyl)-4-oxobutanoic acid **3** was obtained by aluminum chloride mediated Friedel-Crafts acylation of acetanilide **1** with succinic anhydride **2**. Next, acidic hydrolysis of acetamide functionality in compound **3** with concentrated hydrogen chloride afforded 4-(4-aminophenyl)-4-oxobutanoic acid **4**. Subsequent cyclization of keto acid **4** with hydrazine monohydrate lead to the formation of 6-(4-aminophenyl)-4,5-dihydropyridazin-3(2H)-one **5** which was coupled with 4-chloropyridine hydrochloride in the presence of triethylamine to give access to desired 6-(4-(pyridin-4-ylamino)phenyl)-4,5-dihydropyridazin-3(2H)-one (MCI-154).

Reactions were carried out in flame-dried glassware. Transfer of anhydrous solvents and reagents was accomplished with oven-dried syringes. Dimethylformamide (DMF) was distilled before use from calcium hydride; N-methylpyrrolidone (NMP) was dried over 4 Å molecular sieves. Thin layer chromatography was performed on glass plates pre-coated with 0.25 mm Kieselgel 60 F254 (Merck). Flash chromatography columns were packed with 230–400 mesh silica gel (Silicycle). ^1^H NMR were measured on Agilent/Varian DD2 MR two channel 400 MHz spectrometer and are reported in ppm relative to tetramethylsilane (0.00 ppm) standard; coupling constants (*J*) are reported in Hertz (Hz). Standard notation is used to describe the multiplicity of signals observed in ^1^H NMR spectra: singlet (s), doublet (d), triplet (t), etc. Please see the Supplementary Materials for the NMR spectra of MCI-154 and compounds 3-5.

#### 2.1.1 4-(4-acetamidophenyl)-4-oxobutanoic acid (3) [15]

Flame-dried round bottom flask was charged with anhydrous aluminum chloride (13.350 g, 100 mmol) under nitrogen atmosphere and DMF (2 mL, 30 mmol) was added dropwise at 0 °C. To the stirred reaction mixture heated at 70 °C was added the mixture of acetanilide **1** (1.352 g, 10 mmol) and succinic anhydride **2** (1 g, 10 mmol) in little portions. The stirring was continued for 1 h at 70 °C. The warm reaction mixture was poured into ice (90 g) and conc. hydrochloric acid (6 mL) was added. After 15 min the solid precipitate was collected by filtration, washed with water and dried. Recrystallization from DMF/H_2_O mixture afforded the product as beige crystals (1.291 g, 5.48 mmol, 55% yield). ^1^H NMR (400 MHz, DMSO-*d*_6_) δ = 10.26 (s, 1H), 7.94 (d, *J* = 8.8 Hz, 2H), 7.71 (d, *J* = 8.8 Hz, 2H), 3.19 (t, *J* = 6.3 Hz, 2H), 2.56 (t, *J* = 6.3 Hz, 2H), 2.08 (s, 3H) ppm.

#### 2.1.2 4-(4-aminophenyl)-4-oxobutanoic acid (4)

Into the round bottom flask charged with 4-(4-acetamidophenyl)-4-oxobutanoic acid **3** (3. 102 g, 13.19 mmol) was added conc. hydrochloric acid (14 mL) and the reaction mixture was refluxed for 30 min. After cooling down to room temperature the mixture was diluted with water (28 mL) and the acidity was adjusted to pH= 4 at 10 °C by using aqueous saturated solution of sodium carbonate. The product was collected by filtration,washed with water and dried. The product was obtained as off-white powder (0.869 g, 4.46 mmol, 34% yield). ^1^H NMR (400 MHz, DMSO−d_6_) δ = 7.68 (d, *J*=8.8 Hz, 2H), 6.56 (d, *J*=8.8 Hz, 2H), 6.01 (s, 2H), 3.05 (t, *J*=6.3 Hz, H), 2.50 (t, *J*=6.3 Hz, 2H) ppm.

#### 2.1.3 6-(4-aminophenyl)-4,5-dihydropyridazin-3(2H)-one (5) [16]

In a round bottom flask, 4-(4-aminophenyl)-4-oxobutanoic acid **4** (0.850 g, 4.46 mmol) was dissolved in ethanol (5 mL) and hydrazine monohydrate (0.4 mL, 7 mmol) was added. The reaction mixture was refluxed for 24 h. After cooling down to room temperature, the precipitate was filtered off, washed with ethanol and dried. The product was obtained as brown solid (0. 592 g, 3.13 mmol, 70% yield). ^1^H NMR (400 MHz, DMSO- d_6_) δ = 10.60 (s, 1H), 7.45 (d, *J* = 8.8 Hz, 2H), 6.56 (d, *J* = 8.8 Hz, 2H), 5.46 (s, 1H), 2.82 (t, *J* = 8.1 Hz, 2H), 2.35 (t, *J* = 8.1 Hz, 2H) ppm.

#### 2.1.4 6-(4-(pyridin-4-ylamino)phenyl)-4,5-dihydropyridazin-3(2H)-one (MCI-154) [17]

In a round bottom flask, triethylamine (0.22 mL, 156 mmol) was added to the solution of 6-(4-aminophenyl)-4,5-dihydropyridazin-3(2H)-one **5** (0.592 g, 3. 13 mmol) in NMP (3.2 mL). The reaction mixture was heated to 90 °C followed by addition of 4-Chloropyridine hydrochloride (0.455 g, 3.13 mmol) and stirred at 90 °C for 2 h. Then the mixture was cooled down to 0 °C in ice bath, followed by addition of acetone (40 mL). The resulted precipitate was filtered off, dissolved in water and alkalinized with aqueous 1 M solution of sodium hydroxide. The formed precipitate was purified by silica gel flash column chromatography (eluent: CHCl_3_/MeOH, 10:1) which afforded white solid (0.646 g, 2.43 mmol, 78% yield). ^1^H NMR (400 MHz, DMSO- d_6_) δ = 10.83 (s, 1H), 9.02 (s, 1H), 8.23 (d, *J* = 5.9 Hz, 2H), 7.72 (d, *J* = 8.8 Hz, 2H), 7.23 (d, *J* = 8.8 Hz, 2H), 6.97 (d, *J* = 5.9 Hz, 2H), 2.92 (t, *J* = 8.2 Hz, 2H), 2.43 (t, *J* = 8.2 Hz, 2H) ppm. ^13^C NMR (125 MHz, DMSO- d_6_) δ = 166.9, 150.1, 149.3, 149.0, 141.6, 129.5, 126.9, 118.7, 109.8, 26.0, 21.7 ppm. HRMS (ESI+) *m/z* calcd for C_15_H_15_N_4_O [M+H]^+^: 267.1240, found 267.1241.

### 2.2 Protein sample preparation

Recombinant human cardiac cTnC (residues 1–161) with the mutations C35S and C84S, cTnI (residues 1–77), and cTnI (residues 135–209) were used in this study. The expression and purification of ^15^N-cTnC in *E. coli* were as described previously [18]; the expression and purification of cTnI (residues 1–77) and cTnI (residues 135–209) were as described in recent publications [19], [20]. cTnI switch peptide (residues 147–163) was purchased from GL Biochem (Shanghai) Ltd. Stock solutions of MCI-154, in d_6_-DMSO, were prepared, and the vials containing the solutions were wrapped in aluminum foil to protect the molecules from light-catalyzed degradation. For NMR sample preparations, solid ^15^N-labeled cTnC was dissolved into 500 μL NMR buffer containing 100 mM KCl, 10 mM imidazole, 0.5 mM DSS in 90% H_2_O/10% D_2_O to generate a 450 μM NMR sample. 5 μL of 1 M CaCl_2_ was added to ensure that the protein was Ca^2+^-saturated and the pH was adjusted by 1 M NaOH and 1 M HCl to ~ 6.7. For cTnC-cTnI peptide complexes, a 1:1 molar ratio of solid unlabeled cTnI_1–77_ or cTnI_135–209_ peptide was added to solid ^15^N-labeled cTnC prior to dissolution in buffer.

### 2.3 NMR Spectroscopy

All NMR experiments were run on a Varian Inova 500 MHz spectrometer at 30°C. The spectrometer is equipped with a triple resonance ^1^H,^13^C,^15^N-probe and z-axis pulsed field gradients. Both 1D ^1^H and 2D {^1^H, ^15^N}-HSQC NMR spectra were acquired at every titration point. The dissociation constant (*K*_*D*_) for MCI-154 binding to cTnC•3Ca^2+^ was calculated by plotting chemical shift changes as a function of the ligand-to-protein ratio and then fitting the values to a function using our in-house curve-fitting software, xcrvfit (www.bionmr.ualberta.ca/bds/software/xcrvfit). The function relating the predicted change in chemical shift (Δδ) to total protein (*P*) and total ligand concentrations (*L*) is as follows:

$$\Delta \delta ={\Delta \delta }_{\max}\left[\frac{P+L+K_{D}-\sqrt{{(P+L+K_{D})}^{2}-4\mathrm{PL}}}{2P}\right]$$

where ${\Delta \delta }_{\max}$ is the change in chemical shift expected at 100% saturation and *K*_*D*_ is the dissociation constant for the 1:1 protein-ligand complex. A χ^2^ function measuring the sum of differences between observed and predicted Δδ values was minimized, using *K*_*D*_ and ${\Delta \delta }_{\max}$ as fitting parameters.

### 2.4 Titration of MCI-154 to ^15^N-cTnC•3Ca^2+^

For NMR sample preparation, 4.8 mg of solid ^15^N-cTnC were dissolved into 500 μL NMR buffer containing 100 mM KCl, 10 mM imidazole, 0.5 mM DSS in 90% H_2_O/10% D_2_O to generate a 450 μM NMR sample (protein concentration was estimated based on weight and integration of 1D ^1^H NMR spectroscopy). 5 μL of 1 M CaCl_2_ was added to ensure that the protein was Ca^2+^-saturated and the pH was adjusted by 1 M NaOH and 1 M HCl to ~ 6.7. Aliquots of 1, 1, 1, 1, 0.5, 1.5, 2, 5 μL of 90.2 mM MCI-154 in d_6_-DMSO were added consecutively to the ^15^N-cTnC•3Ca^2+^ sample. The sample was mixed thoroughly with each addition. The change in protein concentration due to volume increase was taken into account for data analyses. The pH changes from ligand additions were compensated by 1 M NaOH or 1 M HCl. Both 1D ^1^H and 2D {^1^H, ^15^N}-HSQC NMR spectra were acquired at every titration point.

### 2.5 Titration of cTnI_147–163_ to ^15^N-cTnC•3Ca^2+^

Since cTnI_147–163_ is insoluble in aqueous solutions, solid cTnI_147–163_ was dissolved in d_6_-DMSO to make a 5 mM stock solution, aliquots of 1, 1, 2, 7, 5, 8, 11, 11, 11, 11, 11, 11, 11, 11 μL were added consecutively to a 500 μL NMR sample of 200 μM ^15^N-cTnC•3Ca^2+^ (NMR buffer conditions are 100 mM KCl, 10 mM imidazole, 0.5 mM DSS in 90% H_2_O/10% D_2_O, and protein concentration was estimated based on weight and integration of 1D ^1^H NMR spectroscopy). The sample was mixed thoroughly with each addition. The change in protein concentration due to volume increase was taken into account for data analyses. The pH changes from ligand additions were compensated by 1 M NaOH or 1 M HCl. Both 1D ^1^H and 2D {^1^H, ^15^N}-HSQC NMR spectra were acquired at every titration point.

### 2.6 Isothermal Titration Calorimetry

Isothermal titration calorimetry (ITC) was carried out on a Malvern VP-ITC MicroCalorimeter at 25 °C (note that the NMR experiments were performed at 30 °C), and data were analyzed using the original ITC analysis software package (MicroCal). 2 mM MCI-154 was titrated into 200 μM purified cTnC protein, both in buffer containing 50 mM imidazole at pH 6.8, 100 mM KCl, 10 mM CaCl_2_, and 1% DMSO. The initial injection volume was 4 μL, followed by 15 μL injections spaced 300 s apart. Data were corrected and fitted to a single binding site model, from which apparent molar reaction enthalpy (Δ*H*), entropy (Δ*S*), and dissociation constant (*K*_D_) were generated.

## 3 Results

### 3.1 MCI-154 binds to the structural C-terminal domain of cTnC

**Fig. 2:**
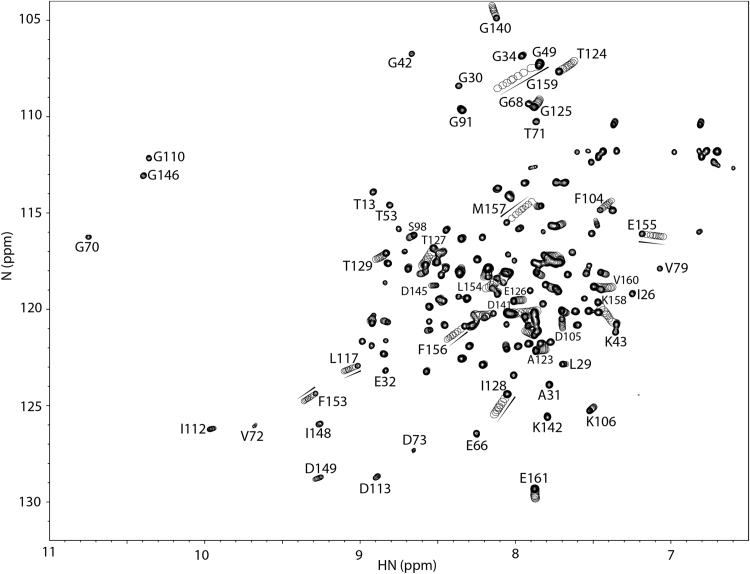
Titration of cTnC•3Ca^2+^ with MCI-154. 2D {^1^H, ^15^N}-HSQC NMR spectra from the backbone amide regions of cTnC at various MCI-154 additions are superimposed, showing the progressive shift of peaks with increasing drug concentrations.

**Fig. 3:**
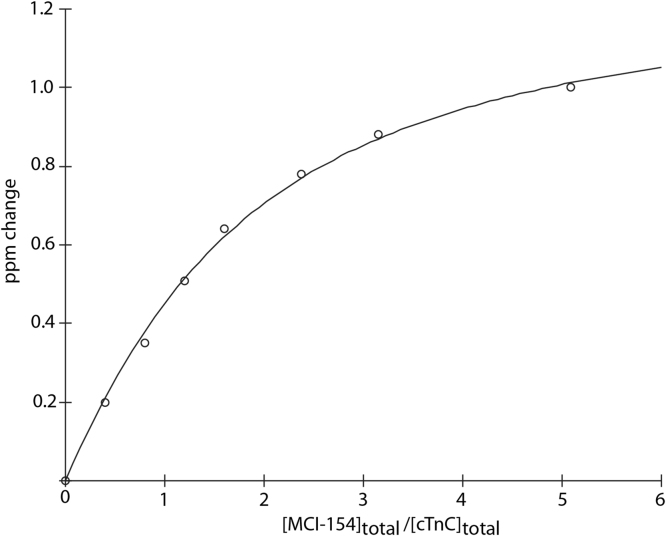
Titration of cTnC•3Ca2+ with MCI-154. The MCI-154-induced chemical shift changes in ppm (Δδ) for each affected residue was calculated as follows: Δδ = [(Δδ_1H_)^2^ + (Δδ_15N_/5)^2^]^1/2^ at every titration point, then averaged for a group of residues (L117, T124, I128, T129, G140, F153, E155, F156, M157, K158, G159, E161) that underwent significant chemical shift changes as shown in Fig. 2. The averaged data were normalized according to (δ_obs_−δ_initial_)/(δ_final_−δ_initial_). The curve was fit as a function of chemical shift perturbation vs. [MCI-154]_total_/[cTnC]_total_. A solid line represents the best fit to the data (K_D_ ~ 0.5 mM).

MCI-154 was titrated into isolated calcium-saturated ^15^N-enriched cTnC, and binding was followed by solution 2D {^1^H, ^15^N} HSQC NMR spectra. Chemical shift changes were observed in residues from the cCTnC domain, but none was observed in the cNTnC domain (Fig. 2). This is somewhat surprising, since one would expect a calcium sensitizer to interact with the regulatory cNTnC domain, which determines the calcium sensitivity of cardiac muscle. (It should also be noted that titration of cTnC with DMSO yields no chemical shift changes.) With each titration point, cCTnC signals progressively move from the unbound state to an MCI-154-bound state without significant broadening, indicating that MCI-154 binding occurs with fast kinetics relative to the NMR frequency differences between free and bound cCTnC states. The MCI-154-induced total chemical shift changes (Δδ) of each affected residue was calculated as follows: Δδ= [(Δδ_1H_)^2^ + (Δδ_15N_/5)^2^]^1/2^, then averaged for a group of residues that underwent significant chemical shift changes. MCI-154 appears to bind to the central hydrophobic cavity of the cCTnC domain (where all small molecules have been found to bind, see Discussion section), as shown by chemical shift perturbation mapping (Fig. 1). The linear movement of the signals as the titration progressed is suggestive of 1:1 stoichiometry. The dissociation constant (*K*_D_) of MCI-154 for cTnC was calculated as described in the Methods section. Fitting of the titration points yields a binding dissociation constant, K_D_, of about 0.5 mM (Fig. 3). This affinity is much weaker than the observed calcium sensitizing activity of MCI-154, which was ~50% active at concentrations of 1–10 μM [21], suggesting that the interaction between MCI-154 and cTnC cannot account for its biological activity.

**Fig. 4:**
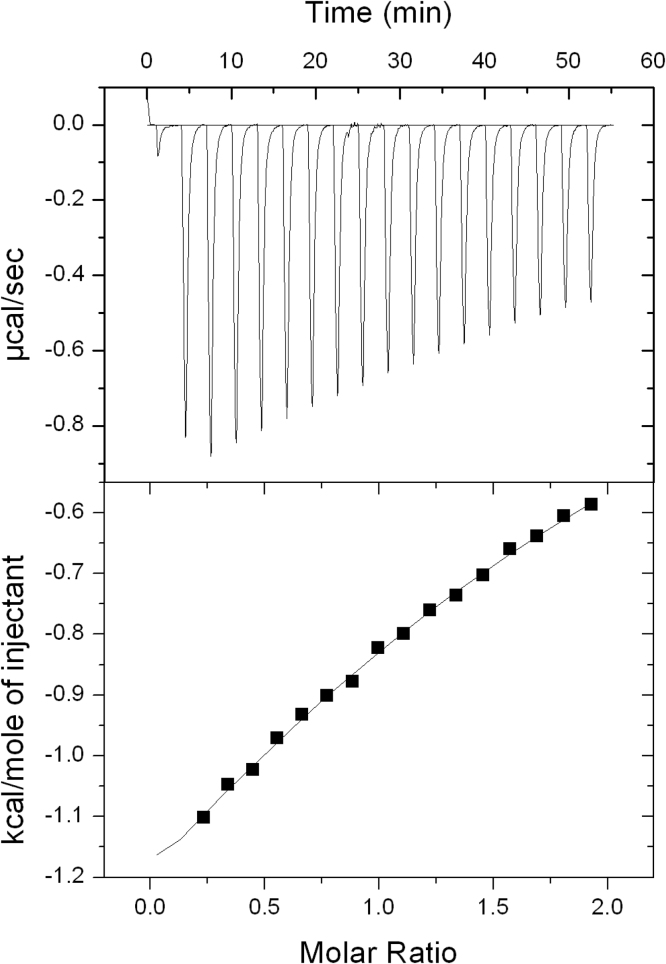
ITC plot of 2.0 mM MCI-154 titrated into 200 μM cTnC protein at 25 °C. The initial injection volume was 4 μL, followed by 15 μL injections spaced 300 s apart. Buffer contained 50 mM imidazole at pH 6.8, 100 mM KCl, 10 mM CaCl_2_, and 1% DMSO.

Isothermal titration calorimetry (ITC) was used to confirm the binding affinity. A K_D_ value of 0.5 mM ± 0.1 mM was obtained, in perfect agreement with the value obtained by NMR. In addition, ΔH of binding was determined to be −2.5 kcal/mol, with ΔS= +6.6 cal/mol K (see Fig. 4). The stoichiometry of binding could not be precisely determined using ITC.

### 3.2 MCI-154 does not bind to cTnC in the presence of cTnI_1–77_

cTnC is strongly tethered to the rest of the cardiac troponin complex by a tight interaction with residues 39–60 of cTnI, which form an amphipathic helix that occupies the large hydrophobic patch in the cCTnC domain [22]. This is the strongest interaction within the troponin complex (nanomolar affinity). We previously demonstrated that residues 19–37 of cTnI interact electrostatically with the regulatory cNTnC domain, and this interaction fixes the positioning of the cNTnC domain relative to cCTnC and the rest of the troponin complex [23].

Full-length cTnI is highly insoluble and does not form a stable soluble complex with cTnC at physiologic pH. We thus prepared a complex of cTnI_1–77_, which is fully soluble, with cTnC. When MCI-154 compound was titrated into cTnC-cTnI_1–77_ complex, no chemical shift changes could be observed in either domain of cTnC. Thus, the interaction between cTnI residues 19–37 and the cNTnC domain did not create a binding site for MCI-154. As well, binding of cTnI residues 39–60 to the cCTnC domain out-competes MCI-154.

### 3.3 MCI-154 does not bind to cTnC in the presence of cTnI_135–209_

cTnC also interacts with cTnI_135–209_ region (see Fig. 1), which comprises all the inhibitory segments that bind to actin to shut down cardiac muscle contraction. It also contains the switch region, cTnI_147–163_, that binds to cNTnC to activate cardiac muscle contraction. Titration of MCI-154 into the cTnC-cTnI_135–209_ complex showed no chemical shift changes. This indicates that MCI-154 does not interact with the cNTnC-cTnI switch region complex. Also, binding of the cTnI_135–209_ peptide to the cCTnC domain out-competes MCI-154.

### 3.4 Binding of full-length cTnC to cTnI_147–163_ switch peptide

**Fig. 5:**
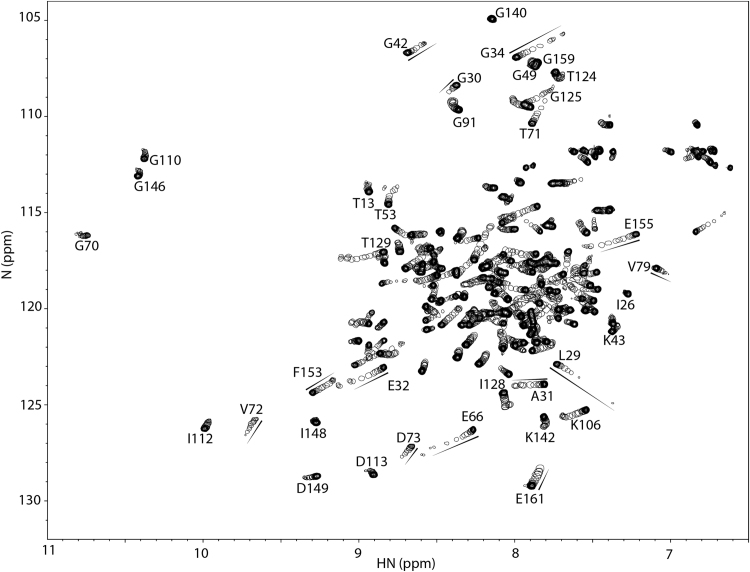
Titration of cTnC•3Ca^2+^ with cTnI_147–163_. 2D {^1^H, ^15^N}-HSQC NMR spectra from the backbone amide regions of cTnC at various peptide additions are superimposed, showing the progressive shift of peaks with increasing peptide concentrations.

It is very well established that the binding of cTnI switch region, cTnI_147–163_, to cNTnC constitutes the key event that triggers cardiac muscle contraction. It was somewhat surprising that binding of cTnI_135–209_ to cTnC should eliminate binding of MCI-154 to the cCTnC domain (see Results Section 3.3 above), since this segment of cTnI was not known to bind the cCTnC domain. To gain more insight into this, we titrated a shorter peptide corresponding to the switch region, cTnI_147–163_, into full-length cTnC. Interestingly, large chemical shift changes were induced in both domains of cTnC (Fig. 5). Given the similar magnitude of chemical shift changes in both domains, one possible explanation is that the cCTnC domain is able to bind the switch region as well as the cNTnC domain. Another possibility is that the cNTnC-cTnI_147–163_ complex interacts with the cCTnC domain. In either case, we have previously shown that when the cCTnC domain is bound to cTnI_37–71_, addition of cTnI_147–163_ into full-length cTnC impacts only the cNTnC domain, and not the cCTnC domain [24]. Apparently, the cCTnC domain is able to bind promiscuously to a variety of peptides and small molecules in the absence of its physiologic binding partner: cTnI residues 39–60.

## 4 Discussion

The binding of the positive inotropic compound MCI-154 to cTnC has not previously been studied by NMR. Previous studies indicated that it directly increased the calcium binding affinity of free cTnC [25], [26], [27], [28]. It is possible that it enhances the affinity of the cCTnC domain by binding to its central hydrophobic cavity, thus stabilizing the calcium-bound open form of the domain. The lack of binding to the cNTnC domain is consistent with our recent study of diphenylamine analogs binding to a cNTnC-cTnI_136–163_ chimeric construct [11]. Bulky substituents in the para position of diphenylamine (as is present in MCI-154, Fig. 1) were not well tolerated. Hence, the positive inotropic effect of MCI-154 in experimental models is most likely related to a mechanism distinct from cTnC binding. MCI-154 has potent inhibitory activity towards type 3 phosphodiesterase, and there is some indication that it may also bind cardiac myosin [29]. These two proteins appear to be among the most promiscuous proteins in cardiomyocytes when it comes to binding small organic molecules.

The structural cCTnC domain of cardiac troponin C is like a “hyper-functioning” version of the regulatory cNTnC domain, binding the primary physiologic target sequence of the cNTnC domain, the switch region of cTnI. It also binds to molecules that cNTnC cannot (like MCI-154, EMD57033, EGCg, and resveratrol), in addition to some that cNTnC can also bind (like trifluoperazine, bepridil, W7, and levosimendan) (for reviews, see [6], [30]). Of the compounds that bind exclusively to the cCTnC domain, MCI-154 binds with a similar affinity (K_D_ ~ 500 μM for MCI-154, compared with 10 μM for EMD 57033 [31], [32], 1120 μM for EGCg [33], and 243 μM for resveratrol [34]). All of these compounds appear to be displaced from the central hydrophobic cavity of the cCTnC domain in the presence of cTnI. We therefore recommend caution in the interpretation of binding and functional studies involving free uncomplexed cTnC [35]. Moreover, it is possible that studies using reconstituted cardiac troponin complex could be influenced by the presence of free cTnC due to stoichiometric excess of cTnC or incomplete incorporation into the troponin complex.
