# Supplementary material for: The calcium sensitizer drug MCI-154 binds the structural C-terminal domain of cardiac troponin C
Source: Biochem Biophys Rep. 2018 Nov 1;16:145–51. doi: 10.1016/j.bbrep.2018.10.012 (PMC6218639; doi:10.1016/j.bbrep.2018.10.012)
Supplement: Supplementary file 2 — Supplementary material. [file mmc2.pdf]

Supplementary Figures: Synthesis of MCI-154 (Scheme-1): the ^{1}H NMR spectra of intermediate compounds **3** (Figure 1), **4** (Figure 2), **5** (Figure 3); and ^{1}H (Figure 4) and ^{13}C NMR (Figure 5) and mass (Figure 6) spectra of final product **MCI-154**.

Figure 1

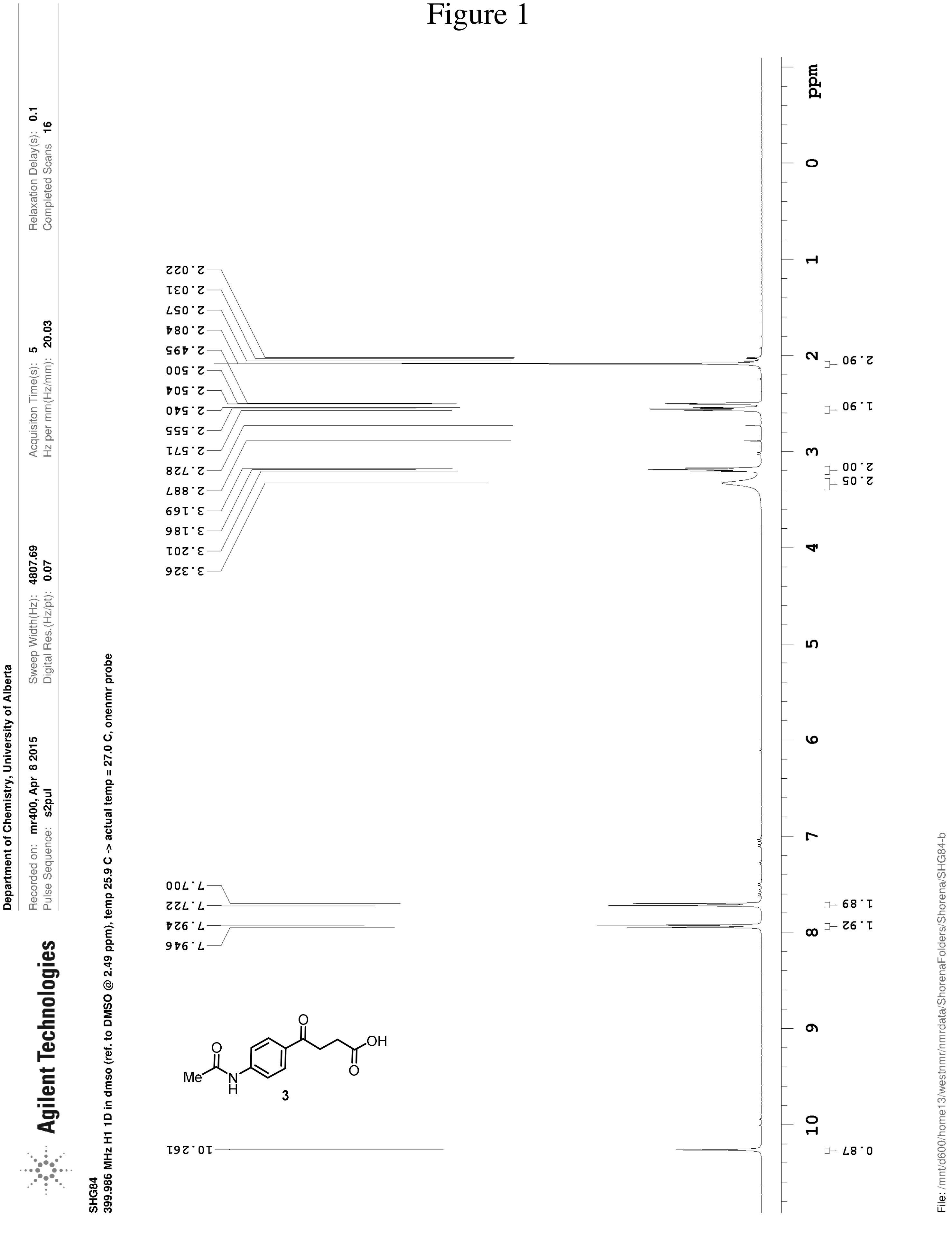
Agilent Technologies
Department of Chemistry, University of Alberta
Recorded on: mr400, Apr 8 2015
Pulse Sequence: s2pul
Sweep Width(Hz): 4807.69
Digital Res.(Hz/pt): 0.07
Acquisiton Time(s): 5
Hz per mm(Hz/mm): 20.03
Relaxation Delay(s): 0.1
Completed Scans 16
SHG84
399.986 MHz H1 1D in dmso (ref. to DMSO @ 2.49 ppm), temp 25.9 C -> actual temp = 27.0 C, onenmr probe
2.022
2.031
2.057
2.084
2.495
2.500
2.504
2.540
2.555
2.571
2.728
2.887
3.169
3.186
3.201
3.326
7.700
7.722
7.924
7.946
10.261
Me
N
H
O
OH
3
2.90
1.90
2.00
2.05
1.89
1.92
0.87
ppm
0
1
2
3
4
5
6
7
8
9
10
File: /mnt/d600/home13/westnmr/nmrdata/ShorenaFolders/Shorena/SHG84-b

Agilent Technologies

Department of Chemistry, University of Alberta

Recorded on: **mr400, Apr 9 2015** Sweep Width(Hz): **4807.69** Acquisiton Time(s): **5** Relaxation Delay(s): **0.1**
Pulse Sequence: **s2pul** Digital Res.(Hz/pt): **0.07** Hz per mm(Hz/mm): **20.03** Completed Scans **16**

SHG86
399.986 MHz H1 1D in dmso (ref. to DMSO @ 2.49 ppm), temp 25.9 C -> actual temp = 27.0 C, onenmr probe

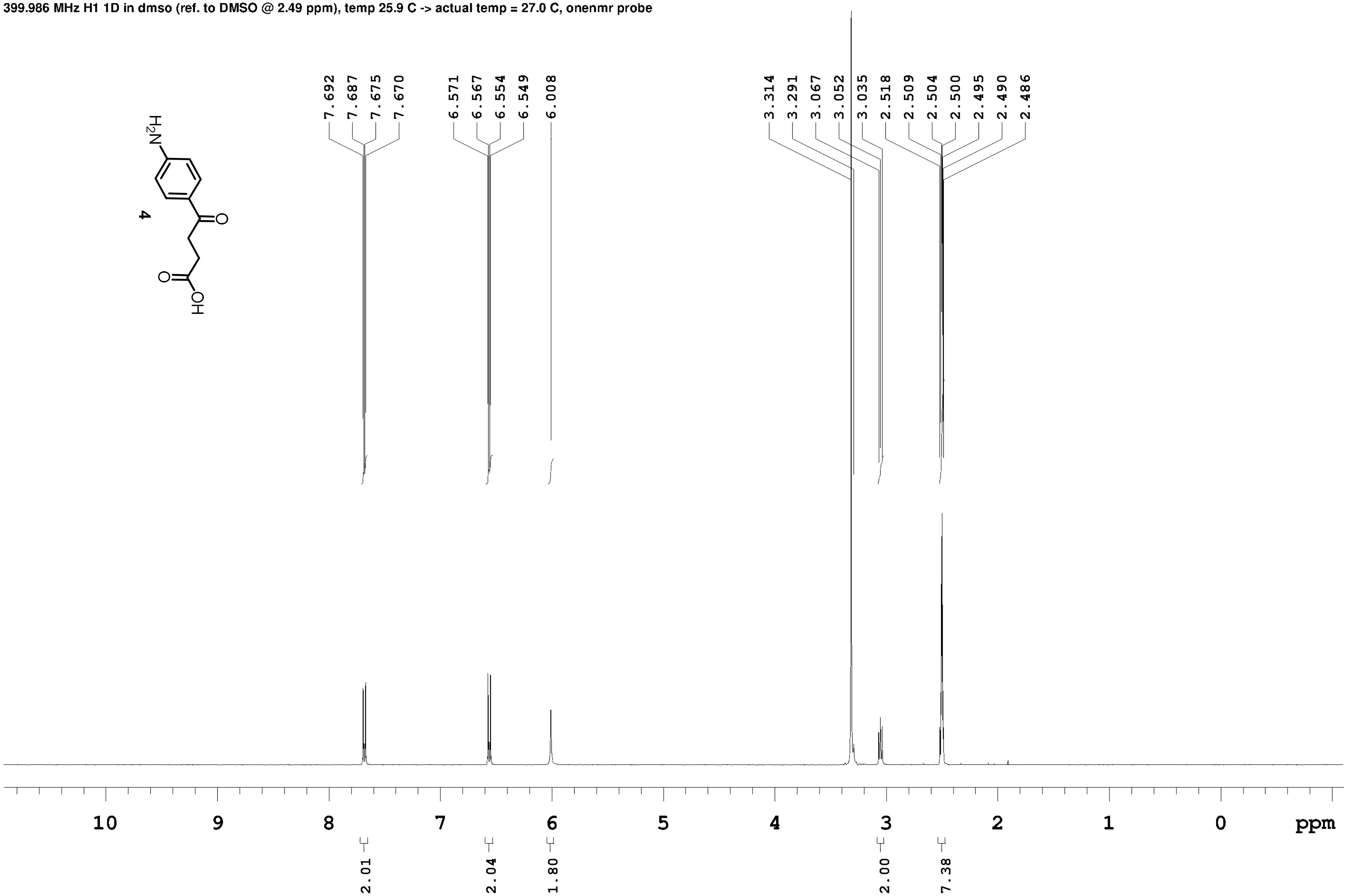

Figure 2

File: /mnt/d600/home13/westnmr/nmrdata/ShorenaFolders/Shorena/SHG86

Agilent Technologies

Department of Chemistry, University of Alberta

Recorded on: **mr400, Apr 13 2015**	Sweep Width(Hz): **4807.69**	Acquisiton Time(s): **5**	Relaxation Delay(s): **0.1**
Pulse Sequence: **s2pul**	Digital Res.(Hz/pt): **0.07**	Hz per mm(Hz/mm): **20.03**	Completed Scans **16**

SHG87?

399.986 MHz H1 1D in dmso (ref. to DMSO @ 2.49 ppm), temp 25.9 C -> actual temp = 27.0 C, onenmr probe

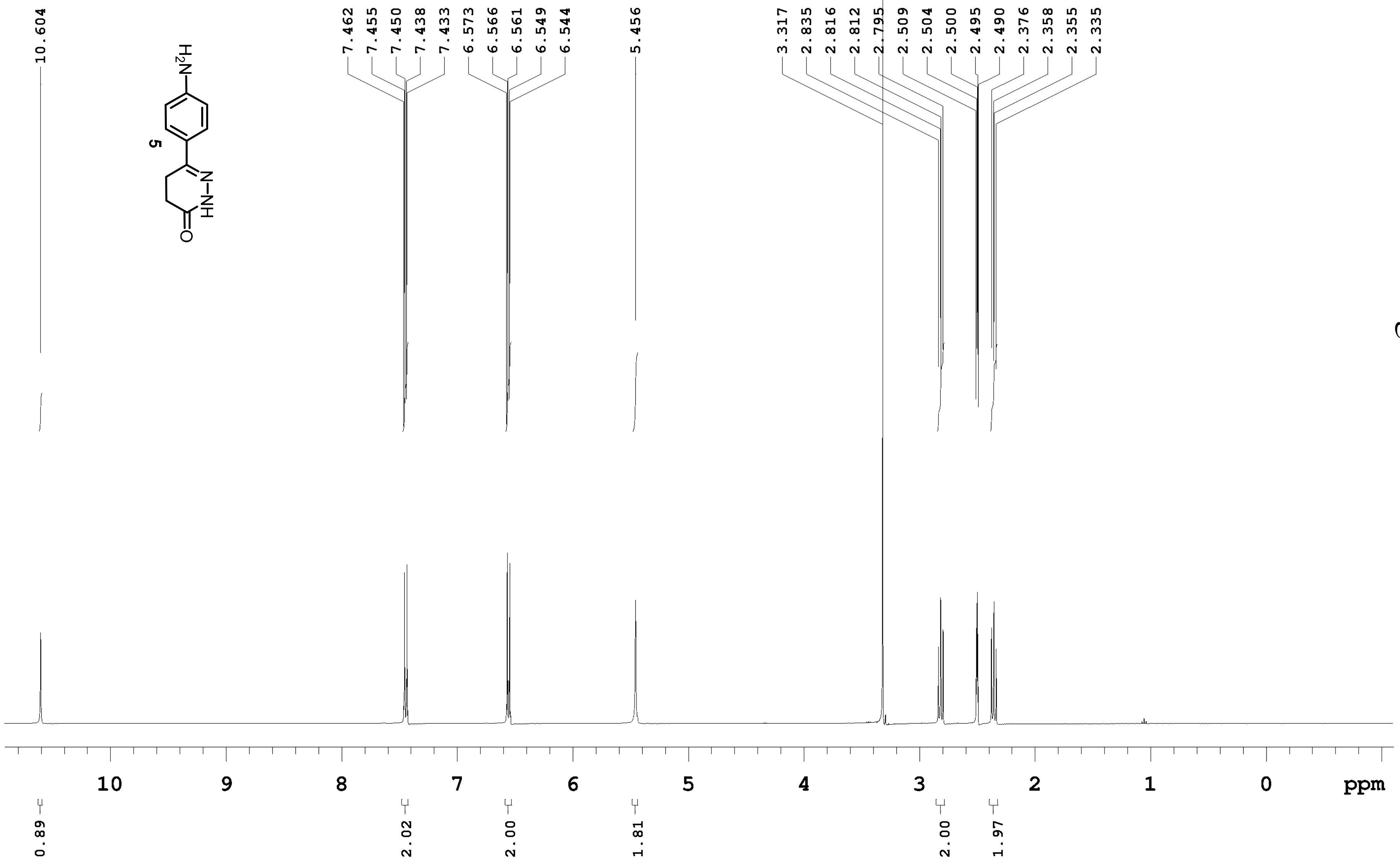

Figure 3

File: /mnt/d600/home13/westnmr/nmrdata/ShorenaFolders/Shorena/SHG87_

Figure 4

Agilent Technologies

Department of Chemistry, University of Alberta

Recorded on: i400, Apr 15 2015
Pulse Sequence: s2pul

Sweep Width(Hz): 4801.92
Digital Res.(Hz/pt): 0.07

Acquisiton Time(s): 4.998
Hz per mm(Hz/mm): 20.01

Relaxation Delay(s): 0.1
Completed Scans 16

SHG88
399.796 MHz H1 1D in dmso (ref. to DMSO @ 2.49 ppm), temp 26.5 C -> actual temp = 27.0 C, autoxdb probe

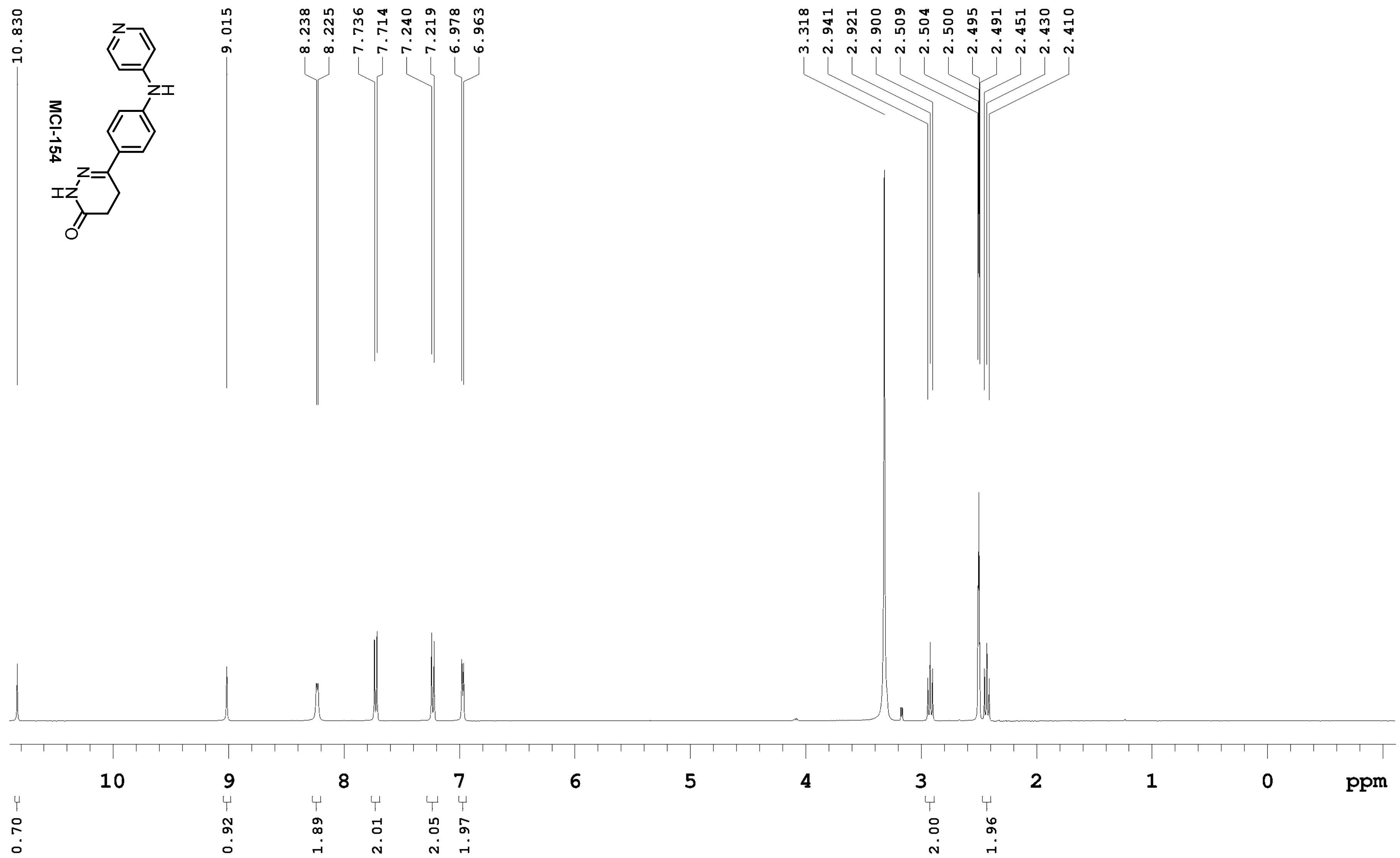

File: /mnt/d600/home13/westnmr/nmrdata/ShorenaFolders/Shorena/SHG88-after_column

Agilent Technologies

Department of Chemistry, University of Alberta

Recorded on: **u500, Apr 15 2015** Sweep Width(Hz): **32894.7** Acquisiton Time(s): **2.5** Relaxation Delay(s): **0.1**

Pulse Sequence: **s2pul** Digital Res.(Hz/pt): **0.25** Hz per mm(Hz/mm): **137.06** Completed Scans **628**

Shorena, SHG88

125.691 MHz C13[H1] 1D in dmso (ref. to DMSO @ 39.5 ppm), temp 27.7 C -> actual temp = 27.0 C, colddual probe

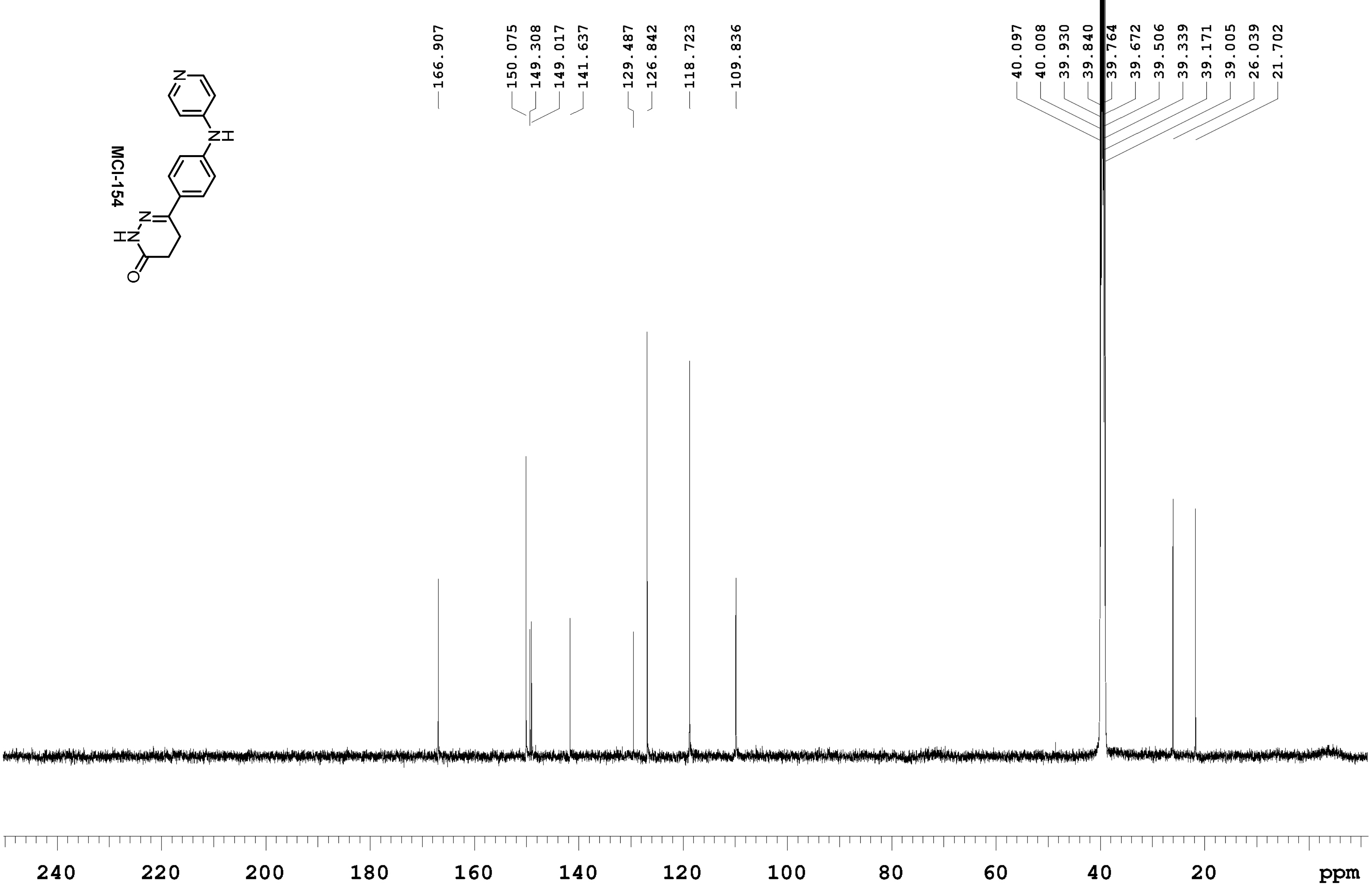

Figure 5

File: /mnt/d600/home13/westnmr/nmrdata/DATA_FROM_NMRSERVICE/Shorena/2015.04/2015.04.15.u5_SHG88_loc1_10.11_C13_1D

shg88 MCI-154 MCI-154

Qualitative Compound Report

University of Alberta

Department of Chemistry

**Comment**	S. Gelozia, West	**Sample Name**	shg 88
**Data File**	15041505.d	**Instrument Name**	oaTOF6220
**Position**	-1	**Operator**	ami
**Acq Method**		**DA Method**	da ami low mass.m

Compound Table

Formula	Mass	Abund
C15H14N4O	266.1168	248437

MS Spectrum

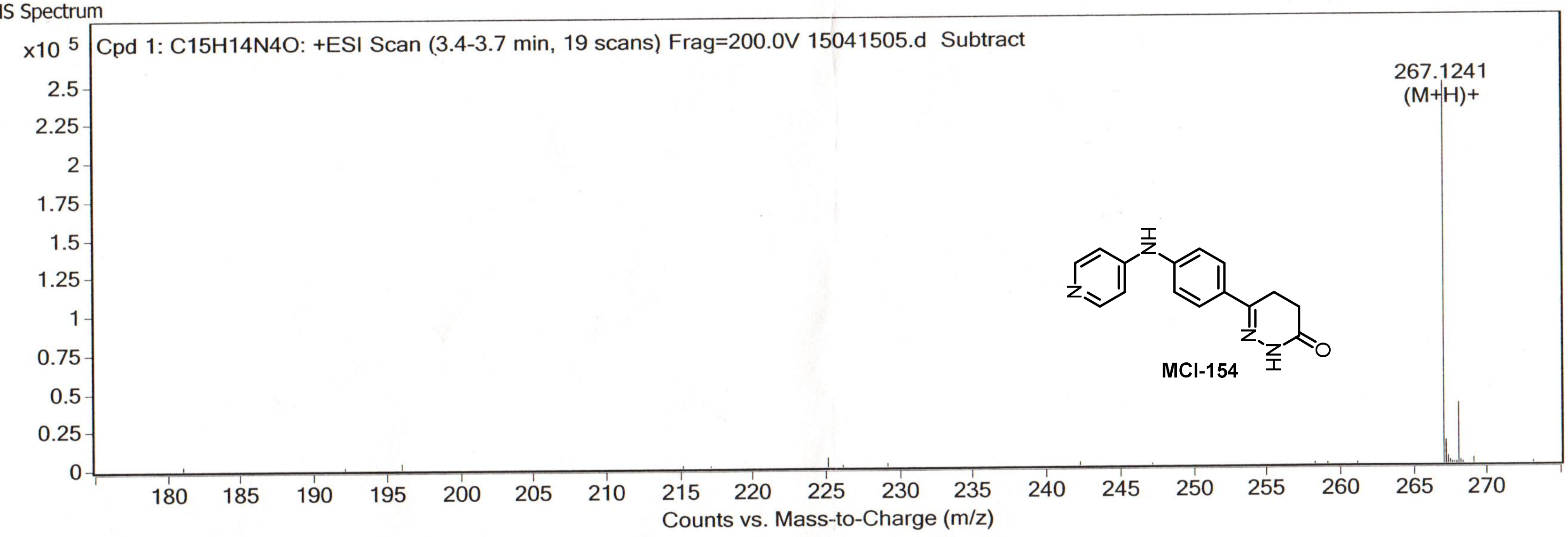

MS Spectrum Peak List

Ion Formula	Ion type	*Measured m/z*	*Calc m/z*	Diff(ppm)	z	Abund
C15 H15 N4 O	(M+H)+	267.1241	267.124	0.32	1	248437

--- End Of Report ---

Figure 6
